# Impact of different pretreatments and attachment materials on shear bond strength between monolithic zirconia restorations and metal brackets

**DOI:** 10.1038/s41598-022-12542-5

**Published:** 2022-05-20

**Authors:** Rebecca Jungbauer, Peter Proff, Daniel Edelhoff, Bogna Stawarczyk

**Affiliations:** 1 Department of Orthodontics, University Medical Centre Regensburg, Regensburg, Germany; 2 Department of Prosthetic Dentistry, LMU Munich, Munich, Germany

**Keywords:** Dental materials, Orthodontics

## Abstract

To investigate the influence of different pretreatment methods, attachment materials and artificial aging on shear bond strength (SBS) between monolithic zirconia and metal brackets. Zirconia substrates were pretreated with silica coated alumina (CoJet) and (1) clearfill ceramic primer plus (CF), (2) RelyX ceramic primer (RXP), (3) Futurabond U (FU). The brackets were bonded with (1) Transbond XT Adhesive (TB), (2) BrackFix Adhesive (BF), (3) bracepaste adhesive (BP). SBS was tested after 24 h, 500 thermal cycles, 90 d at 37 °C with a universal testing machine. SBS values reached from 8.3 to 16.9 MPa. The Weibull moduli ranged between 0.37 (RXP combined with BP after 90 d) and 7.42 (CF combined with TB after 24 h). The pretreatment with FU after 90 d, independent of the attachment material, and RXP with BF resulted in the lowest SBS values 8.3–9.9 MPa, the combination of RXP or CF with TB showed the highest (13.2–16.9 MPa) independent of aging. After FU pretreatment the proportion of ARI 1 and 0 was higher, of ARI 3 lower as after CF and RXP pretreatment. All tested combinations showed sufficiently high SBS values for clinical use. Pretreatment with FU presented the lowest values after 90 days.

## Introduction

For orthodontic treatment with fixed appliances brackets are usually bonded to the enamel surface, using the etch and rinse technique and phosphoric acid^1^. According to the literature shear bond strength (SBS) values between enamel and bracket of 5–10 MPa are recommended^2,3^. In orthodontic adult treatment the bonding of brackets to prosthetically restored teeth can be challenging. There is always the risk of damaging the restoration, but on the other hand accidental bracket loss during the treatment implies a further pretreatment of the restoration, increasing the risk of fractures, but also prolongs treatment time^4^. Nowadays, metal-free restorations are increasingly used, and they are becoming more and more popular due to their good biocompatibility and esthetics^5–7^. Due to the improved esthetics of zirconia, especially 4Y-TZP and 5Y-TZP ceramics, monolithic restorations are used more frequently^8^. Therefore, apart from a reliable bonding strategy to silicate ceramics, the orthodontist also needs a proven workflow for bonding to monolithic zirconia surfaces. Zirconia, in contrast to silicate ceramics, exhibits poor wettability. Since zirconia has no glass components, the wettability and at the same time the surface roughness cannot be influenced by etching with hydrofluoric acid—as known from silicate ceramics^9^. For restorative purposes, an air-abrasion using alumina powder (50 µm grain size) and a low pressure of 0.1 MPa or alternatively using silica coated alumina powder are described to create a suitable surface for adhesive bonding systems to zirconia and veneering materials^10–12^. In addition, the use of an acidic phosphate-containing monomer (e.g. MDP monomer) as a bonding agent is recommended^11^. To date, no gold standard or clinical recommendation exists for conditioning monolithic zirconia restorations when temporary brackets or attachments need to be bonded as part of fixed orthodontic treatment. Although different pretreatment methods have been investigated, most studies focus on the pretreatment methods and include only one attachment material^13–16^. Different attachment materials might also have an influence on the bonding strength between bracket and zirconia surface. The only studies that investigate different attachment materials used bonding materials that are frequently used for prosthetic purposes^17–19^ and not in orthodontic daily routine. Furthermore, the influence of water storage as additional aging regimen has not been considered by any other study. The aim of the in vitro study was therefore to compare the shear bond strength of a metal bracket and monolithic zirconia after air-abrasion using silica coated alumina (CoJet) and application of different adhesive systems using various orthodontic attachment materials and different aging regimens. The following hypotheses were assumed: (1) different pretreatment methods, (2) the use of different light curing attachments materials, (3) different artificial aging methods do not influence SBS values of metal brackets bonded to monolithic zirconia.

## Material and methods

### Specimen preparation

The specimens with a minimum size of 5 × 5 × 3 mm were cut out of zirconia blanks (Ceramill Zolid FX Multilayer, Amann Girrbach, Koblach, Austria) and sintered in the sintering furnace (LHT 02/16, Nabertherm, Lilienthal/Bremen, Germany) according to the manufacturer's instructions. Then, zirconia substrates were embedded in acrylic resin (ScandiQuick A and B, ScanDia, Hagen, Germany) and polished up to P1200 (SiC paper, Struers, Ballerup, Denmark) for 20 s with an automatic polishing device (Tegramin 20, Struers) under permanent water cooling. After ultrasonically cleaning (L&R Transistor/Ultrasonic T-14, L&R, Kearny, NJ, USA) for 60 s in distilled water, the 405 specimens were allocated to one of the 27 test groups (n = 15 per group).

### Zirconia pretreatment and bracket bonding

**Figure 1:**
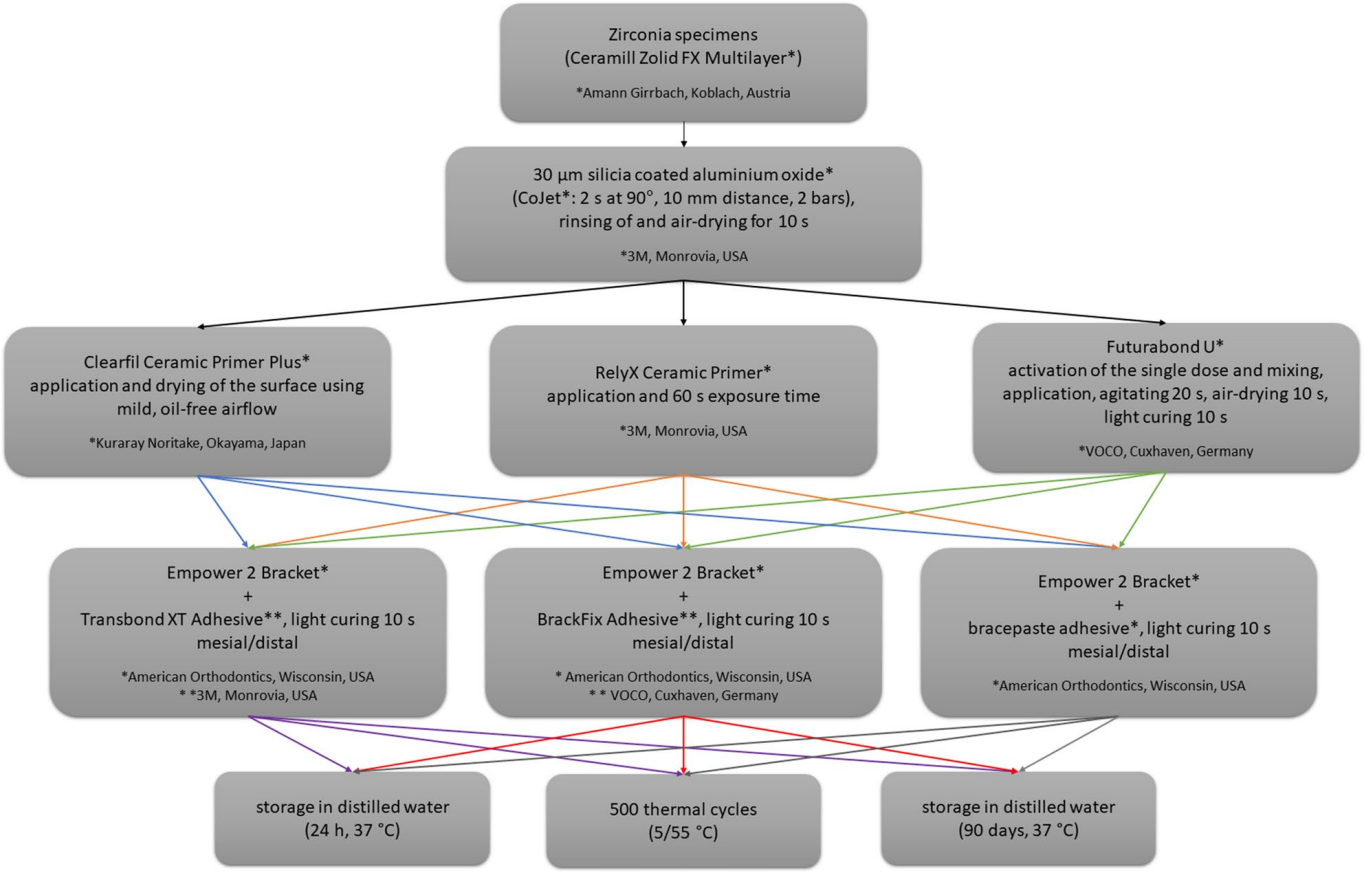
Study workflow. The flowchart shows different pretreatments, attachment materials, and aging regimens of zirconia substrates.

All specimens were photographed with a microscope at 10× magnification (Bresser, Rhede, Germany) to search for possible cracks and afterwards cleaned prior to bonding with a polishing brush (Busch & Co, Engelskirchen, Germany) and pumice/water mixture (40:50 g) at a speed of 3000 rounds per minute moving from left to right as well as up and down for 3 s. The specimens were cleaned with water and air-abraded using silica coated alumina (CoJet Prep and CoJet Sand, both 3 M, Monrovia, USA) for 2 s at 90°, 10 mm distance, 2 bars. After rinsing off the specimens underwent different pretreatments according to the manufacturer’s recommendation (Clearfill Ceramic Primer Plus, CF, Kuraray Noritake, Tokyo, Japan; RelyX Ceramic Primer, RXP, 3 M, Monrovia, USA; Futurabond U, FU, VOCO, Cuxhaven, Germany) and the metal brackets were bonded to the zirconia substrate with different attachment materials (Transbond XT Adhesive, TB, 3 M, Monrovia, USA; BrackFix Adhesive, BF, VOCO, Cuxhaven, Germany; bracepaste adhesive, BP, American Orthodontics, Wisconsin, USA) as detailed presented in Fig. 1. For direct bracket bonding a thin layer of the respective attachment material was applied to the bracket base (Empower 2, American Orthodontics, Wisconsin, USA) and gently pressure was exerted on the bracket to reduce the size of the interface (amount of attachment material) to the possible minimum. All attachment materials were light polymerized, after removing the excess attachment materials around the bracket with a dental probe, for 10 s from the mesial as well as the distal side (1600 mW/cm^2^, Ortholux luminous curing light, 3 M, Monrovia, USA). After bonding, the specimens were directly stored in distilled water and underwent one of the three aging regimens (24 h, 500 thermal cycles, 90 days) as shown in Fig. 1. Detailed information on the materials used is given in Table 1.

**Table 1:** Manufacturer and composition of the luting materials applied in the present study.

Materials	Manufacturer	Composition
TransbondXT adhesive (TB)	3 M	Silane treated quartz, bisphenol-A-diglycidyl ether dimethacrylate, bisphenol-A-bis(2-hydroxyethyl ether) dimethacrylate, silane treated silica, Diphnyliodonium hexafluorophosphate
BrackFix (BF)	VOCO	Bisphenol A-glycidyl methacrylate, bisphenol A ethoxylated dimethacrylate
Bracepast (BP)	American Orthodontics	10-Methacryloyloxydecyl-Dihydrogenphosphate, tetramethylene dimethacrylate, diphenyl(2, 4, 6-trimethylbenzoyl)phosphine oxide
Clearfill ceramic primer plus (CF)	Kuraray Noritake	Ethyl alcohol, 3-methacryloyloxypropyltrimethoxysilane, 10-methacryloyloxydecyl-dihydrogenphosphate
Futurabond U (FU)	VOCO	2-hydroxyethyl methacrylate, bisphenol A-glycidyl methacrylate, hexanedioldimethacrylate, acidic adhesive monomer,urethandimethacrylat
RelyX ceramic primer (RXP)	3 M	Ethyl alcohol, water, methacryloxypropyltrimethoxysilane

### Shear bond strength testing

**Figure 2:**
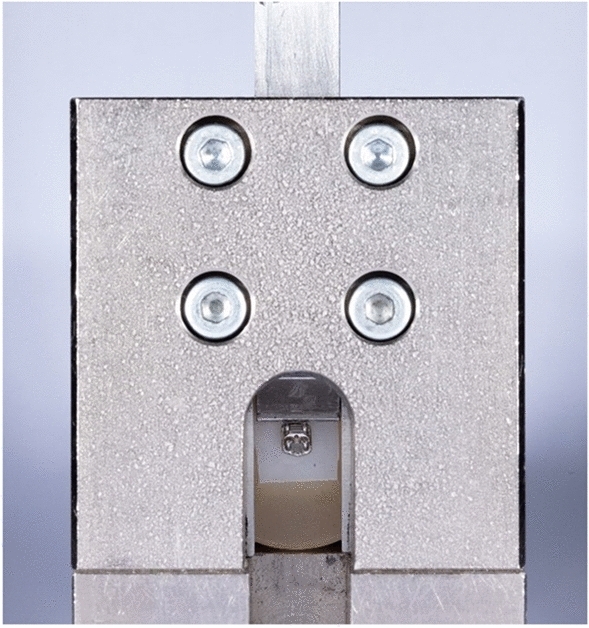
Experimental setup for the SBS measurements. Zirconia substrate with the metal bracket is placed in a special test apparatus. The force was applied in occlusal-gingival direction and the maximum force was recorded.

Shear bond strength testing was carried out at room temperature (23 °C) and all specimens were wetly stored at room temperature for 1 h prior to testing. The specimens were dried carefully with air and placed in the universal testing machine (RetroLine, Zwick/Roell, Ulm, Germany) in a special test apparatus (Fig. 2), before applying a compressive force perpendicularly to the zirconia substrate and in an occluso-gingival direction until debonding of the bracket. A crosshead speed of 1 mm/min was used. The recorded maximum force (F) and the area of the bracket base (A), that was provided by the manufacturer, were used to calculate SBS (R) using the formula:

$$R({\text{N/mm}}^{2} ) = \frac{{F \left( {\text{N}} \right)}}{{A \left( {{\text{mm}}^{2} } \right)}}$$

Afterwards, all specimens were photographed with a microscope at 10× magnification (Bresser, Rhede, Germany) to evaluate the adhesive remnant index (ARI) and look for possible cracks. ARI was determined according to^20^: 0 = no remaining attachment material (AM) on the zirconia, 1 = less than 50% remaining on the zirconia, 2 = more than 50% remaining on the zirconia, 3 = 100% AM remaining on the zirconia.

### Statistical methods

For statistical analyses IBM SPSS Statistics 25 (IBM, Armonk, NY, USA) was used. A global univariate ANOVA test with partial eta-squared $\eta_{p}^{2}$ was performed to determinate the influence of the pretreatment methods, attachment materials and aging regimens on SBS values. Distribution of the data was checked using the Shapiro–Wilk-test. Since the assumption of normal distribution was violated (more than 5% of the tested groups), the data were analyzed non-parametrically using Kruskal–Wallis tests followed by Dunn-Bonferroni post-hoc tests and Bonferroni correction of the *p* values. The distribution of ARI was checked for significant differences using Chi^2^ tests. *p* values of *p* < 0.05 were considered as statistically significant. Weibull module was determined using the maximum likelihood estimation method and 95% confidence interval to be able to predict failure probability^21^.

### Ethical approval

Not applicable, because this article does not contain any studies with animal or human subjects.

## Results

The median of the SBS values ranged between 8.3 and 16.9 MPa (Table 2). SBS values were more influenced by the pretreatment methods ($\eta_{p}^{2}$ = 0.113, *p* < 0.001) and attachment material ($\eta_{p}^{2}$ = 0.110, *p* < 0.001), rather than by aging regimens ($\eta_{p}^{2}$ = 0.019, *p* = 0.025).

**Table 2:** Medians and interquartile ranges (IQR) of shear bond strengths values in MPa.

Pretreatment	Aging	TB			BF			BP		
		Median [IQR]	Min	Max	Median [IQR]	Min	Max	Median [IQR]	Min	Max
CF	24 h	14.9^a AB 1^ [2.9]	10.0	18.0	10.8^b A 1^ [2.6]	7.5	17.8	11.5^ab A 1^ [5.2]	7.9	19.7
FU		11.5^a C 2^ [2.7]	9.7	21.4	10.2^a B 1^ [2.1]	5.9	14.6	11.6^a D 1^ [3.6]	8.4	19.0
RXP		15.6^a D 1^ [6.0]	8.7	30.5	13.5^ab D 1^ [4.1]	7.7	30.8	10.7^b E 1^ [3.5]	7.9	26.1
CF	500 TC	15.6^c A 3^ [5.2]	10.7	22.8	12.9^cd A 2^ [4.9]	4.3	26.8	10.5^d A 2^ [5.1]	7.8	23.5
FU		12.0^b C 4^ [1.8]	7.2	13.6	11.3^b B 2^ [4.2]	8.5	14.6	10.6^b B 2^ [2.1]	7.4	16.2
RXP		15.2^c D 3^ [5.2]	9.1	22.0	11.1^c D 2^ [6.3]	8.5	19.1	11.8^c C 2^ [4.4]	7.4	22.2
CF	90 d	13.2^e B 5^ [3.5]	8.2	18.3	10.5^e A 3^ [3.8]	8.0	22.1	11.8^e A 3^ [6.2]	8.1	22.1
FU		9.9^c C 5^ [2.3]	6.1	22.7	8.3^c C 4^ [3.1]	4.2	11.8	8.7^c B 4^ [4.2]	0.0	14.1
RXP		16.9^d D 6^ [8.1]	12.3	28.8	9.6^e E 3^ [3.2]	7.5	13.9	10.9^e D 3 4^ [6.5]	6.7	20.3

Different pretreatment methods and aging regimens are represented by the rows. Different attachment materials are represented by the columns. Statistical significance of differences (*p* ≤ 0.05) is indicated by superscript letters or numbers. Values that do not differ significantly share the same letter/number. In the rows different uppercase letters indicate significant differences between storage regimens and different numbers between pretreatment methods. Different lowercase letters indicate significant differences in the lines (between attachment materials).

*TB* Transbond XT, *BF* BrackFix, *BP* bracepaste, *CF* Clearfil ceramic primer plus, *FU* Futurabond U, *RXP* RelyX ceramic primer.

### Impact of pretreatment method on the shear bond strength

Within the attachment material TB, FU showed lower initial SBS as well as after thermal cycling than RXP (initial *p* = 0.047, after TC *p* = 0.040) and CF (initial *p* = 0.026, after TC *p* < 0.001). After 90 days water storage, RXP presented higher SBS values compared to FU (*p* < 0.001) and CF (*p* = 0.025). With respect to the attachment material BF, FU showed after 90 days water storage lower values than RXP (*p* = 0.030) and CF (*p* = 0.002). Within BP and after 90 days water stored, lower SBS for FU than for CF (*p* = 0.027) was measured.

### Impact of attachment material on the shear bond strength

Within the pretreatment method CF, TB showed higher initial SBS than BF (*p* = 0.002) and after thermal cycling higher values than BP (*p* = 0.006). With respect to RXP, TB showed higher bond strength compared to BF, regardless on the aging regimen (*p* < 0.001–*p* = 0.043).

### Impact of aging regimen on the shear bond strength

Within TB combined with CF, after 90 days water storage lower SBS than after thermal cycling (*p* = 0.014). Attachment material BF in combination with FU and RXP, 90 days storing in water resulted in lower values than tested initial (FU: *p* = 0.020, RXP: *p* = 0.036) or after thermal cycling (FU: *p* < 0.001, RXP: *p* = 0.027).

### Weibull moduli

The lowest Weibull modulus was observed for BP combined with RXP after 90 days water storage (Table 3). TB combined with CF (initial) or RXP (after thermal cycling) as well as BF combined with FU (after 90 days water storage) showed lower Weibull modulus than TB and BF combined with FU (initial), BP combined with CF (after thermal cycling) and BP combined with all pretreatment methods after 90 days water storage.

**Table 3:** Weibull moduli. Calculated according to the maximum likelihood estimation method with 95% confidence interval for each group separately.

Pretreatment	Aging	TB		BF		BP	
		Weibull module	95% CI	Weibull module	95% CI	Weibull module	95% CI
CF	24 h	7.42	(4.67;13.54)	5.44	(3.19;9.26)	4.57	(2.67;7.78)
FU		3.72	(2.17;6.33)	3.88	(2.27;6.61)	5.04	(2.95;8.57)
RXP		6.64	(3.83;11.12)	5.09	(2.98;8.66)	4.89	(2.86;8.32)
CF	500 TC	5.30	(3.10;9.01)	2.65	(1.54;4.51)	4.05	(2.37;6.89)
FU		4.45	(2.60;7.58)	4.58	(2.68;7.79)	4.52	(2.65;7.69)
RXP		7.28	(4.27;12.39)	6.67	(3.91;11.34)	6.00	(3.51;10.20)
CF	90 d	5.31	(3.11;9.03)	4.66	(2.73;7.92)	3.90	(2.28;6.62)
FU		4.63	(2.71;7.88)	7.17	(4.20;12.20)	3.88	(2.27;6.60)
RXP		4.34	(2.54;7.39)	4.09	(2.39;6.97)	0.37	(0.20;0.64)

*TB* Transbond XT, *BF* BrackFix, *BP* bracepaste, *CF* Clearfil ceramic primer plus, *FU* Futurabond U, *RXP* RelyX ceramic primer.

### Adhesive remnant index (ARI)

**Figure 3:**
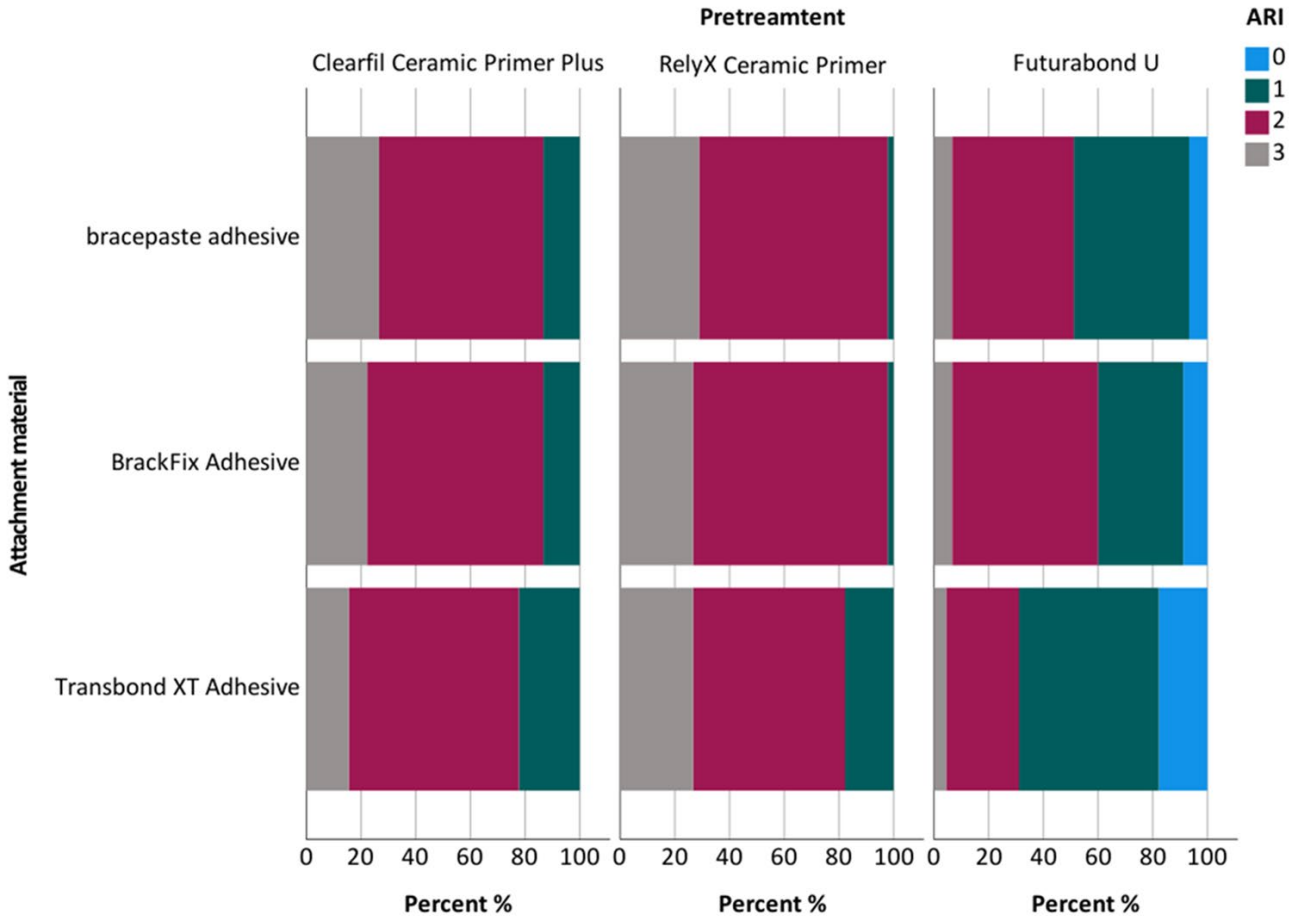
Distribution of ARI. The percentage of the rated ARI (0–3) within each pretreatment and attachment material group is given.

There were differences considering the distribution of the ARI within the different attachment material groups (Chi^2^ < 0.001). In the TB group an ARI of 2 was predominantly observed after pretreatment with CF and RXP (62.2%, 55.6%). After the pretreatment with FU an ARI of 1 was more frequently determined (51.1%). In the BF, as well as the BP group an ARI of 2 was the most frequent after pretreatment with RXP, CF and FU (BF: 71.1%, 64.4%, 53.3%; BP: 68.9%, 60.0%, 44.4%). After FU pretreatment in all three groups the proportion of an ARI 1 and 0 was higher, of an ARI 3 lower as after CF and RXP pretreatment (Fig. 3). No cracks in the ceramic were detected in the zirconia before pretreatment nor after bracket debonding.

## Discussion

To the best of the authors' knowledge, this study is the first to compare different pretreatment methods, attachment materials and aging regimens for bonding brackets to monolithic zirconia including a strategy that was in-vitro proven to be effective for pretreating different types of silicate ceramics.

The first hypothesis “different pretreatment methods do not influence the SBS” was rejected. Within the TB group the application of FU resulted in lower SBS values initially and after 500 TC. After 90 days the combination of RXP and TB showed higher SBS values as FU or CF with TB. In the BF and BP group SBS values after FU pretreatment were lower compared to BF/RXP and BP/CF after 90 days. For restorative purposes the general recommendation in the literature to obtain a reliable adhesion to zirconia includes surface air-abrading with either alumina powder or silica coating (CoJet)^22^ and the application of a silane containing 10-methacryloyloxydecyl dihydrogen phosphate (10-MDP), as the phosphate group can react chemically with the zirconia^23^. Lee et al. reported clinically sufficient SBS values between ceramic brackets and monolithic zirconia after air-abrading with Al_3_O_2_ and pretreatment with different zirconia adhesives but also a universal adhesive containing 10-MDP. In contrast, the pretreatment with a metal primer, not containing 10-MDP, resulted in insufficient SBS values^24^. In the present study, three different types of pretreatment methods were used: CF containing the classical MDP monomer but also a silane. The application of CF showed reliable SBS values which is in accordance with other studies^16,25^. As further pretreatment a classical silane RXP was investigated. The idea was to have a method that would result in reliable SBS values for all types of ceramics. Resulting from a previous study the combination of CoJet and RXP was shown to be effective for bonding brackets to different types of silicate ceramics^26^. The present findings indicate also consistent adhesion using this combination when brackets are bonded to zirconia substrates and is in accordance with the findings of other studies^19,27^ although they used a ceramic instead of a metal bracket. A different study also found sufficiently high SBS values using the combination of CoJet and RXP, although the SBS values were lower and very close to the lower limit of the recommended values^28^. In general, the literature is very controversial as some studies found that air-abrasion and silane application results in sufficient SBS^19,29^ and others reported contrary findings^30^.

The third pretreatment was a universal adhesive (FU). Especially after 90 d of wet storage FU showed in comparison to the other methods lower SBS values. In a prosthetic investigation the combination of air-abrasion followed by CF pretreatment and Panavia F2.0 resulted in comparable tensile bond strength values as followed by FU combined with DuoCem after 5000 thermal cycles^23^. Therefore, further investigations are necessary here. Although other studies apply the corresponding adhesive (bonder) of the attachment material as an additional step of pretreatment^24,28,31^, the present and previous results^29^, indicate that this extra step seems to be mandatory.

The second hypothesis (“different light curing attachment materials do not influence SBS”) was also rejected as the combination of CF/TB showed higher SBS values than CF/BF after 24 h and CF/BP after 500 TC. Within the RXP group the application of TB resulted in higher SBS as BF. In general TB is the most frequently used attachment material in orthodontic SBS studies. There is only very little data on other attachment materials. The combination of alumina powder air-abrasion, CF and Panavia F2.0 showed higher SBS values as in the present study, whereas the use of RelyX U200 instead of Panavia F2.0 was comparable to TB^18^. Nevertheless, in a clinical setting it is of interest to the orthodontist to have a reliable attachment material that can be generally used to bond to enamel but also to artificial surfaces such as ceramic. Neither Panavia F2.0, nor RelyX U200 were developed for orthodontic purposes and are due to their lower viscosity compared to TB, BF or BP not the first choice for direct bracket placement. There is one other study that compared SBS values of TB and BF but in a different context as brackets were bonded to bovine enamel, but they also found similar SBS values between metal brackets and either TB or BF^32^. In accordance with this study, Cetik et al. found the combination of CF and BF to be a suitable pretreatment for zirconia substrate to bond metal brackets^16^. Nevertheless, they found almost double as high SBS values compared to our results. This could be caused by the fact that they treated the zirconia surface with Al_3_O_2_ and applied an additional layer of BrackFix primer or due to the differences in the study setting since they used a crosshead speed of 0.5 instead of 1.0 mm/min. To the best knowledge of the authors there is no other study using bracepaste as attachment material for bracket bonding. Although there were some differences considering SBS values, all attachment materials showed sufficiently high SBS values for clinical use.

The third hypothesis (“different artificial aging methods do not influence SBS”) again was rejected. The pretreatment with CF and application of TB showed lower SBS values after 90 days than after 500 TC. 90 days of storage also reduced SBS of BF/FU and BF/RXP compared to the initial values. In the present study SBS values were tested 24 h after bonding and additionally after two different aging regimens. Thermal cycling is supposed to test the resistance of the adhesion against temperature fluctuations that may cause mechanical stress, volumetric changes or microleakage at the bonding area^33^. In the present study, thermal cycling did not have an influence on SBS. As recommended by the DIN 13990:2017-04 specimens underwent 500 thermal cycles^34^. In contrast to prosthetic restorations brackets need to bond to the teeth only for a limited time period of approximately 2 years^35^, but on the other hand there must be a reliable adhesion throughout the whole multibracket treatment to avoid prolonged treatment times^36^ and undesired tooth movement if teeth are not fixed to the wire. Therefore, including aging regimens into orthodontic SBS testing seems to be of major importance, although this aspect still not considered in many orthodontic SBS studies^1,29,37,38^. Despite of thermal cycling, SBS was tested after 90 days of wet storage at 37 °C. Although wet environment can have an influence on SBS values as is can cause a degradation of fillers and weaken the attachment material or hydrolysis^33^, this kind of aging regimen was only considered in two further orthodontic SBS studies^26,39^. Within the FU groups SBS values were lower for all the different attachment materials after 90 days of wet storage and the combination of FU/BF and FU/BP showed the lowest median SBS values within the study, but values were still in the range of the required 5–10 MPa^2,3^.

In other studies, zirconia was pretreated using alumina powder resulting in reliable SBS values^16,25^. The approach of the present investigation was a to find a clinical pretreatment method that could be used for all type of ceramics. When silicate ceramics are pretreated with alumina powder there is a higher risk to cause fractures and cracks within the ceramic as with silica coating (CoJet) due to the geometry of the particles^10^. Therefore, the CoJet system was used for pretreatment in accordance with a previous study^26^.

With respect to Weibull modulus, the combinations with the adhesive systems containing MDP-monomers showed trends to higher values than a silane. A high Weibull module indicates good bond strength reliability. However, since orthodontic adhesions usually do not have to withstand longer than 2 years, this is a factor that can possibly be neglected. However, further investigations are necessary here.

In all three attachment material groups the distribution of the ARI was different when FU was applied in comparison to CF and RXP as there was a higher percentage of ARI 1 and 0. In the other groups an ARI of 2 was predominant. Many authors interpret an ARI of 2 or 3 as favorable as the risk of implementing cracks into the ceramic is reduced when the fracture occurs in the interface between bracket and attachment material. On the other hand, all the attachment material needs to be removed from the ceramic surface with a drilling device, which again can be a risk for damaging the restoration. Regardless of this consideration the results of the ARI do not reflect clinical reality. The force that is applied to debond a metal bracket in a clinical setting is very different from the force in the standardized testing device. In-vitro the force is only applied in one direction (parallel) from occlusal to gingival. Clinically the brackets or wings are usually squeezed with a special debonding plier, straight cutter or similar. As even the use of different pliers results in significant differences in terms of ARI distribution^40^, the results of in-vitro ARI should be interpreted with great caution as their clinical relevance seems to be very limited.

### Limitations

The results of in-vitro SBS studies always need to be interpreted with caution and are very often criticized. In terms of the method, micro-tensile bond strength tests seem to be favorable compared to shear bond strength as they reduce for example the stress concentration^41–43^. Nonetheless, the shear bond strength set-up in the orthodontic research, especially when brackets are bonded for testing, is closer to clinical conditions, but is also less complex and less expensive. However, there is a strong need to follow standardized protocols in term of study setting insofar as possible and include aging regimens as standard procedure to create a reliable tool for comparative measurements as a basis for further clinical research.

## Conclusions

Within the limitations of the study the following conclusions can be derived:

- All investigated pretreatment methods and attachment materials showed sufficiently high SBS values.
- After 90 days of wet storage the pretreatment with FU presented lower SBS values.
- The previously investigated method (CoJet/CF) for bonding to silicate ceramics, can also be used for bonding to zirconia.

## Data Availability

Data will be provided upon reasonable request.
